# Evolution of Patient-Reported Outcome Measures After Surgery for Degenerative Cervical Spine Disease: Do 1-year PROMs Reflect Longer Term Outcomes?

**DOI:** 10.1227/neu.0000000000003886

**Published:** 2025-12-19

**Authors:** Victor Gabriel El-Hajj, Cecilia Kjaerbeck, Victor E. Staartjes, Erik Edström, Adrian Elmi-Terander

**Affiliations:** 1 Department of Clinical Neuroscience, Karolinska Institutet, Stockholm, Sweden;; 2 Machine Intelligence in Clinical Neuroscience and Microsurgical Neuroanatomy (MICN) Laboratory, Department of Neurosurgery, University Hospital Zurich, Zürich, Switzerland;; 3 Capio Spine Center Stockholm, Löwenströmska Hospital, Stockholm, Sweden;; 4 Department of Medical Sciences, Örebro University, Örebro, Sweden

**Keywords:** PROMs, Patient-reported outcome measures, Cervical spondylosis, Spine surgery

## Abstract

**BACKGROUND AND OBJECTIVES:**

Patient-reported outcome measures (PROMs) are today's gold standard outcomes in surgery for degenerative cervical spine disorders. Long-term outcome research may be limited by missing data at later follow-up time-points. Previous studies on lumbar disorders suggest that no significant changes to PROMS occur after 1-year postsurgery. However, no previous study has examined the evolution of PROMS after surgery for degenerative cervical spine disease. We hence aimed to study the evolution of PROMs at 1, 2, and 5 years of postoperative follow-up in patients with cervical spondylosis treated for degenerative radiculopathy or myelopathy.

**METHODS:**

A total of 6467 patients (4785 radiculopathy and 1682 myelopathy) undergoing surgery for cervical spondylosis between 2006 and 2018 in Sweden were included. Prerequisites for inclusion were PROMs at 1 and 2 or 5 years. The evolution of PROMs over time, the correlation between 1-year PROMs and 2- or 5-year PROMs and the predictive capability of 1-year PROMs were assessed.

**RESULTS:**

No significant changes in PROMs beyond 1-year postoperatively could be observed among both degenerative radiculopathy and myelopathy cohorts (*P* ≥ .05). Significant (*P* < .0001) moderately strong to strong correlations were found between 1-year and 2- or 5-year PROMs (Pearson's R ranging from 0.56 to 0.85). On univariable analysis, 1-year PROMs were found to be strong predictors of long-term outcomes (*P* < .0001).

**CONCLUSIONS:**

After surgery for cervical spondylosis with either degenerative radiculopathy or myelopathy, PROMs remained stable beyond 1 year, with minimal changes occurring thereafter. 1-year PROMs correlated with and served as a good proxy for predicting 2- and 5-year PROMs. This suggests that on a population level, 1-year PROMs are sufficient to assess longer term outcomes.

## Graphical abstract

**Figure:**
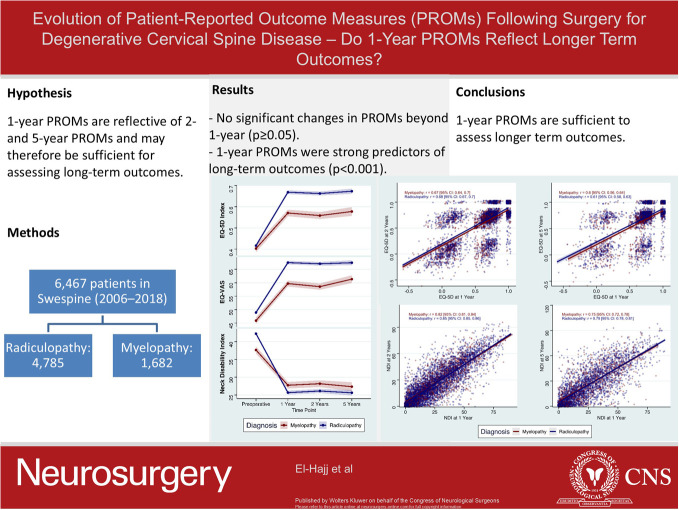

**ABBREVIATIONS:**
ANOVA: analyses of variance
EQ VAS: EuroQol Visual Analogue Scale
EQ5D index: EuroQol 5-Dimension Index
NDI: neck disability index
PROM: patient-reported outcome measures.

Extended follow-up periods are often favored in clinical research, as they provide more comprehensive insights into the long-term efficacy, sustainability, and dependability of medical interventions. These expectations are frequently reinforced by journal guidelines, which commonly require long-term outcome data to support publication.^1^

Nonetheless, consistently collecting long-term data can be challenging due to the logistical burden, financial costs, and frequent issues such as missing data or loss of patient contact over time.

Studies on degenerative lumbar^2-4^ and traumatic cervical spine surgery^5,6^ have shown that outcomes tend to level off approximately 1 year postoperatively. Analogous research focused on degenerative cervical spine disease is lacking. We hypothesized that patient-reported outcome measures (PROMs) after surgery for degenerative cervical spine disease would similarly remain stable beyond the 1-year mark, irrespective of the presentation with either radiculopathy or myelopathy.

Using data from the Swedish Spine Registry (Swespine), which includes PROMs at multiple postoperative intervals, this study set out to determine whether outcomes reported at 1 year could predict results at 2 and 5 years. The analysis was conducted separately for patients who underwent cervical spine surgery due to radiculopathy or myelopathy.

## METHODS

### Study Database

Prospectively collected data from the Swedish spine registry (Swespine) was used to conduct this retrospective observational study. Swespine is Sweden's national quality registry for spine surgery, managed by the Swedish Society of Spinal Surgeons.^5-11^ The registry collects data through surgeon-completed forms and patient questionnaires designed to record patients' subjective assessments of their health status. PROMs are recorded preoperatively and at 1, 2, and 5 years postoperatively for all included patients. The study was approved by the Swedish Ethical Review Authority (Diarienummer: 2020-00193, 2021-04773). Informed consent was not required as per Swedish law regarding retrospective studies.

### Study Population and Design

Individuals surgically treated for cervical spondylosis in Sweden between 2006 and 2018 were identified in the Swespine registry. Only patients with postoperative PROMs at 1 and, either 2 or 5 years, were included. A total of 6467 patients were included, where 4785 were treated for degenerative radiculopathy and 1682 for myelopathy, as reported by the operating surgeons.

### Outcome Variables

PROMs included the EuroQol 5-Dimension Index (EQ5D index), EuroQol Visual Analogue Scale (EQ VAS), and the neck disability index (NDI).

EQ5D assesses health-related quality of life, through 5 principal domains: mobility, personal care, daily activities, pain/discomfort, and anxiety/depression. The EQ-5D Index summarizes responses across 5 dimensions (mobility, self-care, usual activities, pain/discomfort, and anxiety/depression) into a single health utility value, ranging from −0.56 (worst imaginable health) to 1.00 (best imaginable health).

The EQ VAS records the patient's self-rated health on a vertical visual analog scale ranging from 0 (“worst imaginable health state”) to 100 (“best imaginable health state”). It provides a quantitative measure of overall health perception. Finally, the NDI is a condition-specific questionnaire used to measure a patient's neck pain and resulting disability. It consists of 10 items evaluating pain intensity, personal care, lifting, reading, headaches, concentration, work, driving, sleeping, and recreation. The NDI was presented as a percent (%) value where higher values indicate higher disability.

### Statistical Methods

Descriptive statistics were used to describe the baseline characteristics of the population. Continuous variables were expressed as means with 95% CIs, whereas categorical ones were reported as frequencies (n) and percentages (%).

To illustrate the changes in PROMs over time, linear graphs of the means with error bars representing SEs were used.

To assess the evolution of PROMs at 1, 2, and 5 years postoperatively, repeated analyses of variance (ANOVA) were performed. The assumption of normality was tested using the Shapiro-Wilk test to determine if the data were suitable for ANOVA. The assumption of sphericity was checked using Mauchly's test. The test will show if the variability of the differences between time points is roughly equal. Otherwise, the results of ANOVA can be misleading. If sphericity was violated, the Greenhouse-Geisser correction was used to adjust the degrees of freedom in the ANOVA. Post hoc comparisons between time points were performed using the Bonferroni correction.

Scatter plots were created to illustrate the changes in 2- or 5-year PROM scores relative to 1-year PROM scores. To assess the correlation between 1-year PROMs and 2- or 5-year PROMs, Pearson's correlation coefficient was calculated. Using the correlation coefficient (*r*) correlations were interpreted as follows: 0.0 to 0.3 as weak, 0.3 to 0.7 as moderate, and 0.7 to 1.0 as strong. To assess the statistical significance of the association corresponding *P*-values were determined. To evaluate the predictive value of 1-year PROMs, linear regression models were used to predict 2-and 5-year PROMs using 1-year PROMs as the independent variable. The regression outputs included beta coefficients (B), SEs, R^2^ values, and *P*-values to assess the magnitude of association and model fit.

All statistical analyses were conducted in R version 4.4.3 (R Foundation for Statistical Computing). The significance threshold was set at *P* ≤ .05.

### Availability of Data and Materials

The Data sets used and/or analyzed during the current study are available from the corresponding author upon reasonable request.

## RESULTS

### Baseline Characteristics

**FIGURE 1.**
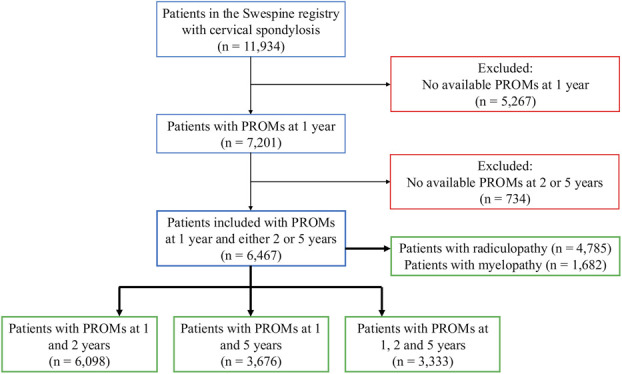
Flowchart. PROM, patient-reported outcome measures.

The entire cohort included 6467 patients, where 4785 were treated for radiculopathy and 1682 for myelopathy (Figure 1). The mean age was 54.4 years (95% CI: 52-62), with a higher age in the myelopathy group compared with radiculopathy (61.4 vs 52.0 years). Men and women were equally represented in the study population (Table 1). The mean body mass index was 26.8 (95% CI: 26-27) with no significant difference between groups. Patients with a history of previous spine surgery constituted 10%, with no major difference between the radiculopathy and myelopathy groups.

**TABLE 1.** Baseline Characteristics of the Included Cohort

Characteristic	Overall, N = 6467	Radiculopathy, N = 4785	Myelopathy, N = 1682
Male sex	3319 (51%)	2404 (50%)	915 (54%)
Age	54.41 (52-62)	51.96 (51-52)	61.36 (61-62)
BMI	26.80 (26-27)	26.78 (26-27)	26.87 (26-27)
Smoker	863 (13%)	660 (14%)	203 (12%)
Previous spine surgery	652 (10%)	500 (10%)	152 (9.0%)
Neck pain duration			
<3 mo	189 (3.3%)	110 (2.5%)	79 (5.9%)
3-12 mo	1388 (25%)	1101 (26%)	287 (21%)
1-2 y	1113 (20%)	915 (21%)	198 (15%)
>2 y	2511 (44%)	2001 (46%)	510 (38%)
No neck pain	463 (8.2%)	189 (4.4%)	274 (20%)
Missing	803	469	334
Arm pain duration			
<3 mo	247 (4.4%)	155 (3.6%)	92 (6.8%)
3-12 mo	1810 (32%)	1413 (33%)	397 (29%)
1-2 y	1368 (24%)	1098 (25%)	270 (20%)
>2 y	1888 (33%)	1497 (35%)	391 (29%)
No arm pain	361 (6.4%)	160 (3.7%)	201 (15%)
Missing	793	462	331
Pain medication use			
Daily	2848 (44%)	2301 (48%)	547 (33%)
As needed	1804 (28%)	1413 (30%)	391 (23%)
None	1815 (28%)	1071 (22%)	744 (44%)
Opioid use	718 (11%)	605 (13%)	113 (6.7%)
Maximal distance walked (m)			
<100	533 (9.5%)	173 (4.0%)	360 (27%)
100-500	615 (11%)	362 (8.5%)	253 (19%)
500-1000	750 (13%)	569 (13%)	181 (14%)
>1000	3710 (66%)	3175 (74%)	535 (40%)
Missing	859	506	353
Fine motor skills impairment	3988 (71%)	2893 (68%)	1095 (82%)
Missing	885	532	353
Type of admission			
Elective	6183 (96%)	4690 (98%)	1493 (89%)
Nonelective	284 (4.4%)	95 (2.0%)	189 (11%)
Approach			
Anterior	4839 (75%)	3905 (82%)	934 (56%)
Posterior	1628 (25%)	880 (18%)	748 (44%)
Procedure			
ACDF	4335 (67%)	3587 (75%)	748 (44%)
Laminectomy	951 (15%)	211 (4.4%)	740 (44%)
Foraminotomy	677 (10%)	669 (14%)	8 (0.5%)
ACCF	261 (4.0%)	92 (1.9%)	169 (10%)
Arthroplasty	243 (3.8%)	226 (4.7%)	17 (1.0%)
No. of levels operated	1.657 (1.4-2.2)	1.48 (1.4-1.5)	2.15 (2.1-2.2)
Total length of hospital stay (d)	2.52 (2.1-3.7)	2.17 (2.1-2.2)	3.53 (3.4-3.7)

ACCF, anterior cervical corpectomy and fusion; ACDF, anterior cervical diskectomy and fusion; BMI, body mass index.

Neck pain for more than 2 years was reported in 44% of the entire cohort, 1 to 2 years in 20%, 3 to 12 months in 25%, and less than 3 months in 3.3%. No neck pain was reported in 8.2%. Arm pain for more than 2 years was reported in 33% of the entire cohort, 1 to 2 years in 24%, 3 to 12 months in 32%, and less than 3 months in 4.4%. No arm pain was reported in 6.4%.

Daily use of pain medication was reported in 44% and opioid use in 11% of the overall cohort.

Most of the patients (66%) were able to walk more than 1000 m. Patients with myelopathy had more walking difficulties with 27% unable to walk more than 100 m. Overall, most of the patients had fine motor skills impairment (71%) with a higher prevalence in the myelopathy compared with the radiculopathy group (82% vs 68%).

Most patients (96%) were admitted on elective basis, with a higher rate of nonelective admissions in the myelopathy compared with radiculopathy group (11% vs 2%).

Anterior procedures included anterior cervical diskectomy and fusion (67%), corpectomy (4%), and arthroplasty (3.8%), whereas posterior ones included laminectomy (15%) and foraminotomy (10%). On average, patients underwent surgery on 1.66 levels, with the radiculopathy group having a lower number of levels operated compared with the myelopathy group (1.45 vs 2.15 levels).

Overall, the mean length of hospital stay was 2.53 days, with shorter stays being observed in the radiculopathy group compared with the myelopathy group (2.15 vs 3.53 days).

### Evolution of PROMs Over Time

**FIGURE 2.**
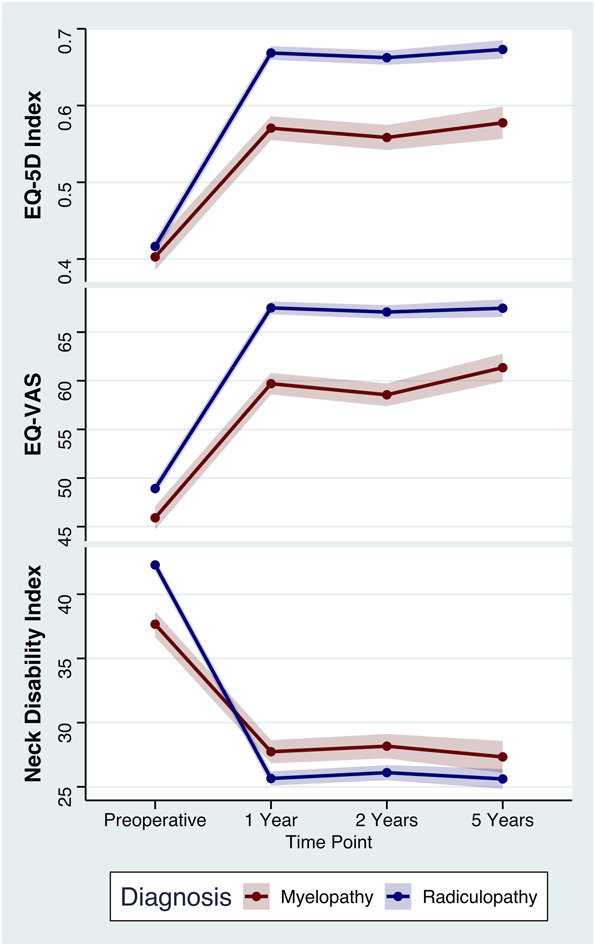
Line graph with 95% CIs showing the evolution of patient-reported outcome measures (preoperative and at 1, 2, and 5 years postoperatively) in patients with cervical spondylosis with either radiculopathy or myelopathy. EQ VAS, EuroQol Visual Analogue Scale; EQ5D index, EuroQol 5-Dimension Index.

Patients in both radiculopathy and myelopathy groups reported significant improvements compared with preoperative baseline levels, regarding EQ5D index, EQ VAS, and NDI (Figure 2). A plateau was observed beyond 1-year of follow-up. On repeated measures ANOVA, no statistically significant variation in PROMs could be detected over time. Comparing values at 1, 2, and 5 years yielded nonsignificant *P*-values in both radiculopathy (EQ5D index: *P* = .145; EQ VAS: *P* = .516; NDI: *P* = .624) and myelopathy groups (EQ5D index: *P* = .313; EQ VAS: *P* = .516; NDI: *P* = .451). Post hoc Bonferroni-adjusted comparisons of within-group differences also failed to identify any statistically significant changes over any of the specific timepoints (*P* ≥ .05).

### Correlation Between 1-year PROMs and 2- or 5-year PROMs

**FIGURE 3.**
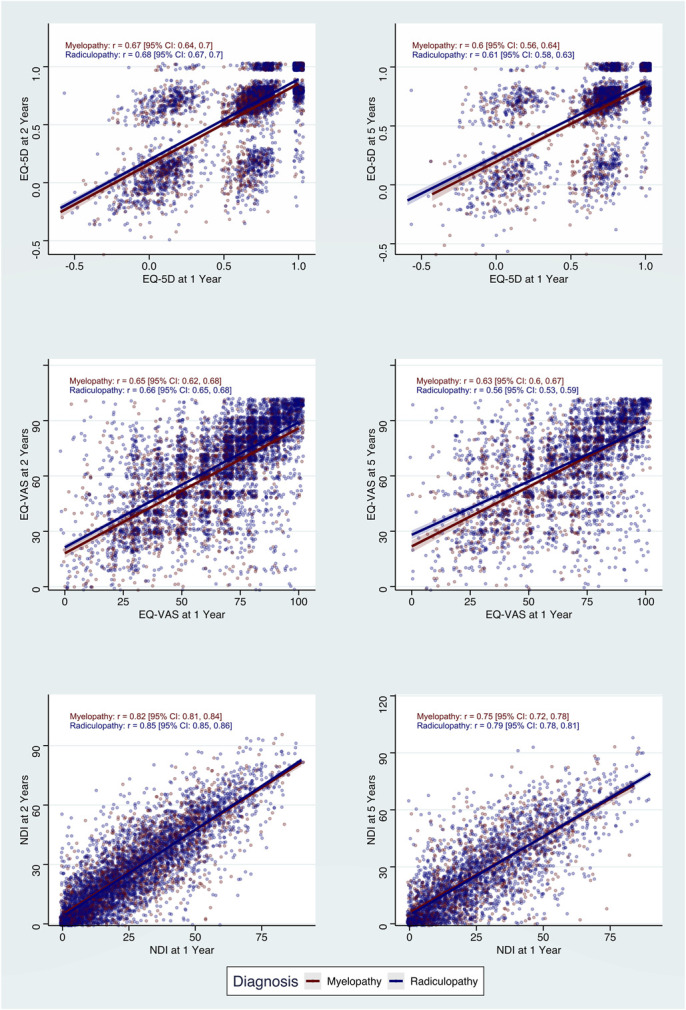
Scatter plot matrix showing the correlation between 1-year patient-reported outcome measures and 2- or 5-year patient-reported outcome measures for EQ5D index, EQ VAS, and NDI in patients undergoing surgery for cervical spondylosis with either radiculopathy or myelopathy. The Pearson correlation coefficients along with their 95% CIs are provided. EQ VAS, EuroQol Visual Analogue Scale; EQ5D index, EuroQol 5-Dimension Index; NDI, neck disability index.

Scatter plots illustrate the concordance between 1- and 2- or 5-year PROM values (Figure 3). The Pearson correlation coefficient revealed a significant (*P* < .0001) and moderately strong, to strong association between 1-year PROMs and 2- or 5-year PROMs. Specifically, 1-year PROMs demonstrated a stronger correlation with long-term outcomes for NDI with Pearson's R ranging from 0.75 to 0.85, as compared with the EQ5D or EQ VAS with Pearson's R ranging from 0.56 to 0.68.

### Predictive Capability of 1-year PROMs

The linear regression models demonstrated that 1-year PROMs were strong and significant (*P* < .0001) predictors of 2- and 5-year PROMs in both the radiculopathy and myelopathy groups (Table 2). Notably, the 1-year NDI showed the highest predictive value, with a strong correlation observed for both 2-year (B = 0.88, R^2^ = 0.73) and 5-year NDI (B = 0.83, R^2^ = 0.63) in the radiculopathy group, and similarly in the myelopathy group (2-year NDI: B = 0.86, R^2^ = 0.68; 5-year NDI: B = 0.80, R^2^ = 0.56). For the EQ5D and EQ VAS, 1-year scores demonstrated moderate to strong predictive ability, with the 1-year EQ5D showing a B-coefficient of 0.70 for 2-year outcomes (R^2^ = 0.47) and 0.63 for 5-year outcomes (R^2^ = 0.37) in the radiculopathy group, and a B-coefficient of 0.69 for 2-year outcomes (R^2^ = 0.45) and 0.64 for 5-year outcomes (R^2^ = 0.36) for the myelopathy group. For EQ VAS, the model for the prediction of 2-year EQ VAS using 1-year EQ VAS showed a B-coefficient of 0.68 for 2-year outcomes (R^2^ = 0.44) and 0.58 for 5-year outcomes (R^2^ = 0.31) in the radiculopathy group, and a B-coefficient of 0.68 for 2-year outcomes (R^2^ = 0.42) and 0.65 for 5-year outcomes (R^2^ = 0.40) for the myelopathy group.

**TABLE 2.** Summary of Linear Regression Models Predicting 2- and 5-Year PROMs Based on 1-Year PROMs

Group	Dependent variable (predictor)	Independent variable (predicted)	Beta-coefficient (B)	SE	R^2^	*P*-value
Radiculopathy	1-year EQ5D index	2-year EQ5D index	0.70	0.01	0.47	<.0001
	1-year EQ5D index	5-year EQ5D index	0.63	0.02	0.37	<.0001
	1-year EQ VAS	2-year EQ VAS	0.68	0.01	0.44	<.0001
	1-year EQ VAS	5-year EQ VAS	0.58	0.02	0.31	<.0001
	1-year NDI	2-year NDI	0.88	0.001	0.73	<.0001
	1-year NDI	5-year NDI	0.83	0.01	0.63	<.0001
Myelopathy	1-year EQ5D index	2-year EQ5D index	0.69	0.02	0.45	<.0001
	1-year EQ5D index	5-year EQ5D index	0.64	0.03	0.36	<.0001
	1-year EQ VAS	2-year EQ VAS	0.68	0.02	0.42	<.0001
	1-year EQ VAS	5-year EQ VAS	0.65	0.03	0.40	<.0001
	1-year NDI	2-year NDI	0.86	0.01	0.68	<.0001
	1-year NDI	5-year NDI	0.80	0.02	0.56	<.0001

EQ VAS, EuroQol Visual Analogue Scale; EQ5D index, EuroQol 5-Dimension Index; NDI, neck disability index; PROM, patient-reported outcome measures.

## DISCUSSION

This nationwide analysis of over 6000 patients undergoing surgery for degenerative cervical spine disease indicates that the main postoperative improvement in PROMs occurs within the first year after surgery. Although significant improvements are shown between preoperative and 1-year postoperative data, a plateau in the improvements of PROMs can be observed after 1 year, with no significant changes beyond this time point. Strong or moderately strong correlations were seen between 1-year PROMs and 2- or 5-year PROMs. A particularly strong correlation was seen for the NDI, whereas slightly weaker correlations were seen with EQ5D and EQ VAS, at longer follow-up times, which could in part be due to interferences caused by age-related health issues developed since index surgery. Furthermore, 1-year PROMs were a strong predictor of long-term outcomes at 2 and 5 years, as indicated by the linear regression models. Despite that, a decent proportion of variance in the 2- and 5-year scores could not explained by the 1-year values as indicated by the R^2^ values. This indicates that although most patients remain stable after 1 year, individual variation continues to exist beyond the 1-year mark.

Both the radiculopathy and the myelopathy groups reported postoperative NDI scores worse than that of the general population.^12^ Preoperatively, the radiculopathy group reported worse NDI scores but showed greater postoperative improvement compared with the myelopathy group. Radiculopathy typically leads to pain and sensory disturbances which can be more rapidly alleviated surgically compared with myelopathy where surgery aims to halt progression of neurological decline.^13^ The observed differences in preoperative scores and degree of improvement between the 2 groups align with findings from previous studies.^14^

Similarly, both groups had postoperative EQ5D index and EQ VAS scores below general population norms.^15^ The myelopathy group demonstrated worse scores both preoperatively and postoperatively. This likely reflects the broader impact of myelopathy on health-related quality of life because it affects multiple domains assessed by the EQ5D index. Because these impairments are often not fully reversible, EQ5D index and EQ VAS improvements are more limited in myelopathy compared with radiculopathy. These findings suggest that a number of patients, particularly those with myelopathy, may fail to return to premorbid health levels despite appropriate surgical intervention.

The lack of significant changes in PROMs at 1, 2, and 5 years postoperatively may reflect a long-term stabilization of symptoms, indicating that no further improvements are usually observed beyond the 1-year mark, and that the recovery achieved during the first year is largely maintained. These results are in accordance with previous findings in the literature.^2-6^

Despite a limited number of studies reporting on the evolution of PROMs in cervical spondylosis, the overall findings seem coherent and consistent. A study of 144 patients with cervical myelopathy from the Norwegian Registry for Spine Surgery conducted additional long-term follow-ups beyond the registry's 1-year data. Outcome measures included NDI as the primary outcome as well as European Myelopathy Scale, EQ5D, and numeric rating scales for headache, neck pain and arm pain. The study reported that patients remained mostly stable in their PROMs after 1-year postoperatively.^16^ Similarly, one study including 630 patients from the Quality Outcome Database, representing centers across the United States, found that after surgery for cervical spondylotic myelopathy, PROMs, including EQ5D index, NDI, and other measures improved for most of the patients within the first year and remained stable thereafter.^17^ Studies analyzing clinician-reported neurological improvement after surgery for degenerative cervical spine disease using the Modified Japanese Orthopaedic Association scale have similarly shown that most of the recovery occurs within the first year.^17-21^ Thus, it is reasonable to expect that PROMs; reflecting patients' subjective experience, would follow the same pattern and also stabilize within the first year after surgery. However, some studies have indicated that between one-quarter and one-third of patients experience deterioration by a decade postsurgery, regarding the Modified Japanese Orthopaedic Association score^22,23^ and the Nurick grade.^24^ It is worth mentioning that these studies involved relatively small cohorts, and that the observed decline may, in part, reflect age related comorbidities or the development of new spinal pathology rather than deterioration of surgical outcomes.

Overall, our findings are in line with the expected patterns of recovery after surgical treatment of degenerative cervical spine disease. In clinical practice, these early PROM scores could act as a basis for decisions regarding treatment and follow-up strategies. The results also indicate that once a patient has reached favorable outcomes at around 1 year after surgery, less frequent monitoring may be indicated. Recognizing the typical trajectory of PROMs can help clinicians identify deviations that may indicate an abnormal or unfavorable outcome requiring further rehabilitative or surgical interventions. In summary, these findings may support clinical decision-making and facilitate more personalized care for patients with degenerative cervical spine disease. This study also raises important considerations about how research on surgical outcomes can be optimized. Defining a timeframe for PROM follow-ups has the potential to enhance research efficiency by reducing unnecessary long-term data collection. Given the resource-intensive nature of maintaining quality registries, setting clear follow-up benchmarks could allow for more efficient allocation of those resources.

### Strengths and Limitations

The strength of this study is the large nationwide sample size of patients with PROMs collected for multiple years. This study is the largest to date analyzing PROMs after surgery for cervical spondylosis. Although the study likely includes a nationally diverse patient population, it is focused exclusively on data from the Swedish population, which may limit its external validity and generalizability internationally. However, comparable findings reported in studies from other countries support the results, indicating their robustness for broader applicability. As reflected by the flowchart (Figure 1), using voluntary questionnaires poses the risk of attrition bias when patients are lost to follow-up. This has the greatest impact on data 5 years postoperatively, where questionnaires have a lower response rate. The use of voluntary questionnaires can also lead to selection bias when some patients may be more prone to report their symptoms, and some less. Groups of patients less engaged with health care or unable to answer questionnaires might be underrepresented. Self-reported symptoms over multiple years also raises the issue of recall bias. However, previous studies on spine registries in the Nordic countries, including Swespine, have shown that loss of follow-up did not lead to significant biases.^25-27^ In addition, although non–significant *P*-values do not inherently rule out the possibility of a meaningful difference, the large sample size of our cohort provided high statistical power. As determined by post hoc sensitivity analyses, even small effect sizes would have reached significance if present. The lack of significant differences across 1-, 2-, and 5-year PROMs therefore strengthens the conclusion that outcomes remain stable beyond 1 year. Finally, a limitation of our study is that we did not establish predefined criteria for comparability of PROMs across time points based on the test–retest reliability of each instrument. Although our analyses demonstrate stability at the group level, this approach does not capture whether individual patients remained within an expected range of variation relative to their 1-year scores. Future studies should incorporate reliability thresholds or minimal clinically important difference values to more precisely characterize individual-level stability over time.

## CONCLUSIONS

In patients undergoing surgery for cervical spondylosis, PROMs remained stable beyond 1 year with minimal changes occurring thereafter. PROMs at 1 year successfully correlated with and served as a good proxy for predicting 2- and 5-year PROMs. This suggests that short term 1-year PROMs are strong predictors of long-term outcomes. Although this relationship is robust at the population level, some degree of individual variability remains inevitable. Therefore, the use of 1-year PROMs in clinical decision-making, guideline creation, and research may be sufficient for reliably estimating final surgical outcomes at a population level.

## References

[1] Ahmad SS Hoos L Perka C Stöckle U Braun KF Konrads C. Follow-up definitions in clinical orthopaedic research: a systematic review. Bone Jt Open. 2021;2(5):344-350. 34044582 10.1302/2633-1462.25.BJO-2021-0007.R1 PMC8168549

[2] Fekete TF Loibl M Jeszenszky D How does patient-rated outcome change over time following the surgical treatment of degenerative disorders of the thoracolumbar spine? Eur Spine J. 2018;27(3):700-708. 29080002 10.1007/s00586-017-5358-2

[3] Parai C Hägg O Lind B Brisby H. Follow-up of degenerative lumbar spine surgery-PROMs stabilize after 1 year: an equivalence study based on Swespine data. Eur Spine J. 2019;28(9):2187-2197. 31041598 10.1007/s00586-019-05989-0

[4] Staartjes VE Siccoli A de Wispelaere MP Schröder ML. Patient-reported outcomes unbiased by length of follow-up after lumbar degenerative spine surgery: do we need 2 years of follow-up? Spine J. 2019;19(4):637-644. 30296576 10.1016/j.spinee.2018.10.004

[5] El-Hajj VG Singh A Blixt S Edström E Elmi-Terander A Gerdhem P. Evolution of patient-reported outcome measures, 1, 2, and 5 years after surgery for subaxial cervical spine fractures, a nation-wide registry study. Spine J. 2023;23(8):1182-1188. 37094774 10.1016/j.spinee.2023.04.014

[6] El-Hajj VG Stenimahitis V Singh A The effect of concomitant spinal cord injury on postoperative health-related quality of life after traumatic subaxial cervical spine injuries: a nationwide registry study. Arch Phys Med Rehabil. 2024;105(6):1069-1075. 38369229 10.1016/j.apmr.2024.01.021

[7] Buwaider A El-Hajj VG MacDowall A Machine learning models for predicting dysphonia following anterior cervical discectomy and fusion: a Swedish Registry Study. Spine J. 2025;25(3):419-428. 39505010 10.1016/j.spinee.2024.10.010

[8] El-Hajj VG Gustafsson MRV Clement B Weight change patterns following surgery for cervical spondylosis in overweight and obese individuals: a nationwide longitudinal study. Eur Spine J. 2025;34(8):3421-3429. 40533577 10.1007/s00586-025-09070-x

[9] El-Hajj VG Staartjes VE Charalampidis A Patient-reported outcome measures and satisfaction after laminectomy for degenerative cervical myelopathy in octogenarians: an observational study from the national Swedish spine registry. Eur Spine J. 2025;34(7):3003-3011. 40347290 10.1007/s00586-025-08890-1

[10] El-Hajj VG Klukowska AM Staartjes VE Minimally clinically important relative change instead of difference following surgery for degenerative cervical radiculopathy: a nationwide study on 5300 patients using prospectively collected data. J Neurosurg Spine. 2025;43(4):464-471. 40749227 10.3171/2025.4.SPINE2598

[11] El-Hajj VG Klukowska AM Staartjes VE, et al. Minimal clinically important difference and relative change in patient-reported outcomes after surgery for cervical spondylotic myelopathy: a nationwide study of 1,700 patients. J Neurosurg Spine. 2025;43(4):464-471. 40637438 10.1227/neu.0000000000003596

[12] Kato S Takeshita K Matsudaira K Tonosu J Hara N Chikuda H. Normative score and cut-off value of the Neck Disability Index. J Orthop Sci. 2012;17(6):687-693. 22895822 10.1007/s00776-012-0276-y

[13] Zuckerman SL Devin CJ. Outcomes and value in elective cervical spine surgery: an introductory and practical narrative review. J Spine Surg. 2020;6(1):89-105. 32309649 10.21037/jss.2020.01.11 PMC7154366

[14] Toci GR Lambrechts MJ Karamian BA Patients with radiculopathy have worse baseline disability and greater improvements following anterior cervical discectomy and fusion compared to patients with myelopathy. Spine J. 2023;23(2):238-246. 36257530 10.1016/j.spinee.2022.10.005

[15] Janssen MF Szende A Cabases J Ramos-Goñi JM Vilagut G König HH. Population norms for the EQ-5D-3L: a cross-country analysis of population surveys for 20 countries. Eur J Health Econ. 2019;20(2):205-216. 29445941 10.1007/s10198-018-0955-5 PMC6438939

[16] Johansen TO Holmberg ST Danielsen E Long-term results after surgery for degenerative cervical myelopathy. Neurosurgery. 2024;94(3):454-460. 37823669 10.1227/neu.0000000000002712 PMC10846761

[17] Zeitouni D Johnson SE Ibrahim S Patient-reported outcome trajectories the first 24 months after surgery for cervical spondylotic myelopathy: a Quality Outcomes Database study. J Neurosurg Spine. 2025;42(4):500-508. 39793008 10.3171/2024.9.SPINE24351

[18] Evaniew N Coyle M Rampersaud YR Timing of recovery after surgery for patients with degenerative cervical myelopathy: an observational study from the Canadian spine outcomes and research network. Neurosurgery. 2023;92(2):271-282. 36637265 10.1227/neu.0000000000002213

[19] Badhiwala JH Witiw CD Nassiri F Efficacy and safety of surgery for mild degenerative cervical myelopathy: results of the AOSpine North America and international prospective multicenter studies. Neurosurgery. 2019;84(4):890-897. 29684181 10.1093/neuros/nyy133 PMC6425462

[20] Pandita N Gupta S Raina P Neurological recovery pattern in cervical spondylotic myelopathy after anterior surgery: a prospective study with literature review. Asian Spine J. 2019;13(3):423-431. 30685954 10.31616/asj.2018.0139 PMC6547403

[21] Moussellard HP Meyer A Biot D Khiami F Sariali E. Early neurological recovery course after surgical treatment of cervical spondylotic myelopathy: a prospective study with 2-year follow-up using three different functional assessment tests. Eur Spine J. 2014;23(7):1508-1514. 24777670 10.1007/s00586-014-3315-x

[22] Dijkman MD van Bilsen MWT Fehlings MG Bartels RHMA. Long-term functional outcome of surgical treatment for degenerative cervical myelopathy. J Neurosurg Spine. 2022;36(5):830-840. 34826817 10.3171/2021.8.SPINE21651

[23] Xu BS Zhang ZL Le Huec JC Xia Q Hu YC. Long-term follow-up results and radiographic findings of anterior surgery with Cloward trephination for cervical spondylotic myelopathy. J Spinal Disord Tech. 2009;22(2):105-113. 19342932 10.1097/BSD.0b013e31816d6579

[24] Sarkar S Rajshekhar V. Long-term sustainability of functional improvement following central corpectomy for cervical spondylotic myelopathy and ossification of posterior longitudinal ligament. Spine (Phila Pa 1976). 2018;43(12):e703-e711. 29068879 10.1097/BRS.0000000000002468

[25] Højmark K Støttrup C Carreon L Andersen MO. Patient-reported outcome measures unbiased by loss of follow-up. Single-center study based on DaneSpine, the Danish spine surgery registry. Eur Spine J. 2016;25(1):282-286. 26208938 10.1007/s00586-015-4127-3

[26] Elkan P Lagerbäck T Möller H Gerdhem P. Response rate does not affect patient-reported outcome after lumbar discectomy. Eur Spine J. 2018;27(7):1538-1546. 29523985 10.1007/s00586-018-5541-0

[27] Ingebrigtsen T Aune G Karlsen ME Non-respondents do not bias outcome assessment after cervical spine surgery: a multicenter observational study from the Norwegian registry for spine surgery (NORspine). Acta Neurochir (Wien). 2023;165(1):125-133. 36539647 10.1007/s00701-022-05453-x PMC9840578

